# Supine-Position Ultrasound-Assisted Arthroscopic Surgery for Elbow Joint Contracture: A Case Report

**DOI:** 10.7759/cureus.46921

**Published:** 2023-10-12

**Authors:** Toru Omodani

**Affiliations:** 1 Orthopedics, Tokyo Advanced Orthopedics, Tokyo, JPN

**Keywords:** elbow dislocation, supine position, arthroscopy, ultrasound-assisted surgery, elbow joint contracture

## Abstract

A 16-year-old male had a right elbow contracture after dislocation. The patient underwent supine-position ultrasound-assisted arthroscopic surgery, combining open removal of the posterior oblique ligament with the medial collateral ligament. The elbow joint flexion angle improved from 120 degrees to 145 degrees, and the extension angle improved from -50 degrees to 0 degrees. The patient returned to sports without recurrence of elbow joint contracture three months after surgery. Supine-positioned ultrasound-assisted arthroscopic surgery for elbow joint contracture is a safe and effective technique that eliminates the need for intraoperative position changes and big skin incisions.

## Introduction

Elbow joint contracture is a debilitating condition characterized by a limited range of motion and functional impairment [1]. The incidence of elbow joint contracture varies, but it is a relatively common condition [2]. Conservative treatment options, such as physical therapy and bracing, can be effective in some cases; however, surgical intervention may be indicated in more severe or persistent cases [3].

Several surgical techniques have been described for the management of elbow joint contracture, including open and arthroscopic approaches [4]. The posterior oblique ligament of the medial collateral ligament is typically removed in the supine position, whereas arthroscopic capsular release is usually performed in the prone position [5]. Few reports exist on supine-position elbow arthroscopy, and those almost always involve the use of an arm positioner [6].

In this case report, we describe a refined surgical technique that combines ultrasound-assisted arthroscopic capsular release in the supine position with open removal of the posterior oblique ligament of the medial collateral ligament, eliminating the need for intraoperative position changes. The patient was informed that data concerning the case would be submitted for publication, and he provided consent.

## Case presentation

A 16-year-old male experienced a restriction in the range of motion of his right elbow joint after dislocation while playing ice hockey. At the time of injury, the patient underwent a reduction for elbow joint dislocation at a nearby clinic. After 3 weeks of immobilization, range of motion exercises were initiated. Rehabilitation failed to improve the range of motion, and the patient was referred to our institution 8 months after the injury. The elbow joint had a fixed flexion deformity of 50 degrees, and it could only flex up to 70 degrees. No ossification of the joint capsule or myositis ossificans was observed in the radiographs. The only abnormal image finding was joint capsule thickening and shortening observed in the magnetic resonance imaging. Therefore, it was believed that the cause of the elbow joint contracture was due to the thickening and shortening of the joint capsule. It was decided to perform open removal of the posterior oblique ligament of the medial collateral ligament and excision of the anterior joint capsule.

**Figure 1:**
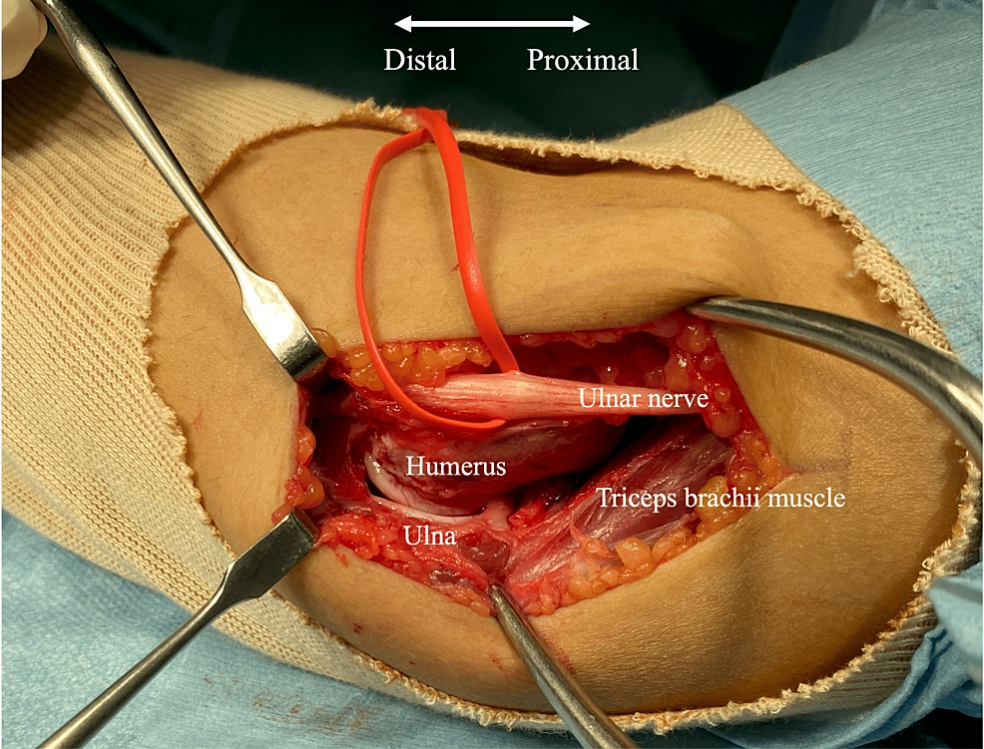
Open procedure for the medial elbow The posterior oblique ligament of the medial collateral ligament was removed with a scalpel, and the posterior joint capsule was detached from the humerus.

Initially, a skin incision was made on the medial side of the elbow joint in the supine position, then the ulnar nerve was identified and protected. The posterior oblique ligament of the medial collateral ligament was removed with a scalpel, and the posterior joint capsule was detached from the humerus (Figure 1). The elbow joint flexion angle improved to 145 degrees. The ulnar nerve was returned to its original position without anterior transposition, and the skin incision was closed.

**Figure 2:**
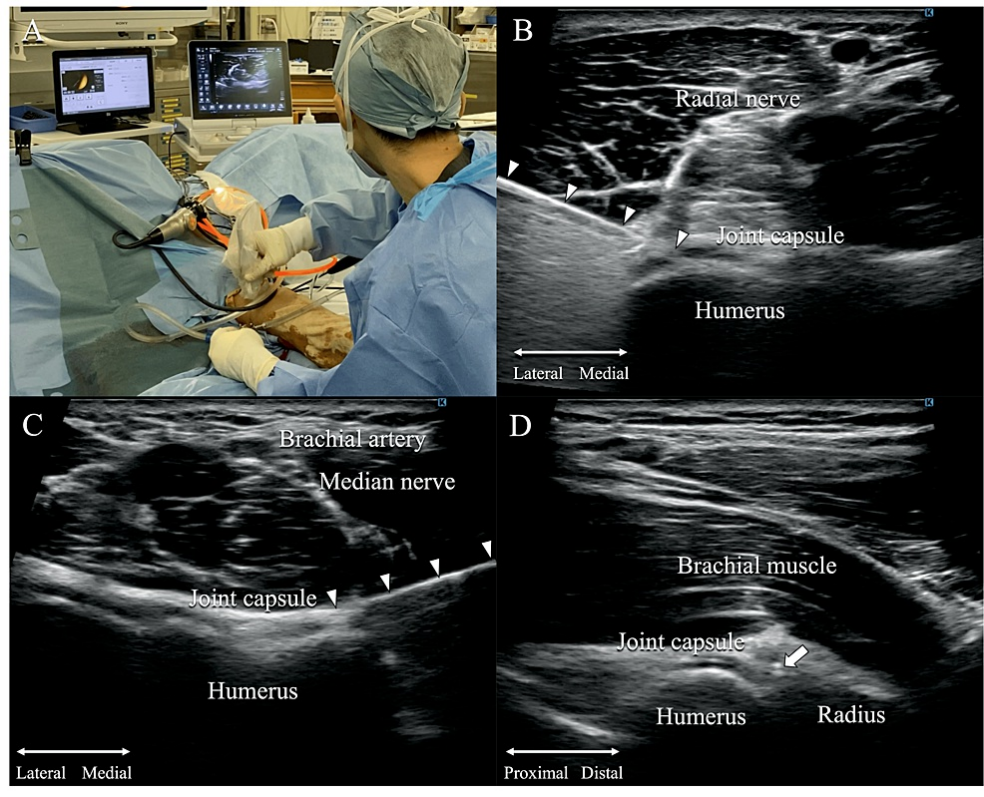
Ultrasound-guided portal making An anterolateral portal was created under ultrasound guidance (A). An arthroscopy device was inserted confirming the nerves and blood vessels (B: anterolateral portal, C: anteromedial portal). The ultrasound image confirmed that the tip of the arthroscopy device was inserted between bone and joint capsule in long axis view (D). Arrowhead: arthroscopy device. Arrow: the tip of the arthroscopy device.

Next, an ultrasound probe covered with a sterile sleeve was placed on the anterior aspect of the elbow joint, and the positions of the nerves and blood vessels were confirmed (11-MHz linear probe, SONIMAGE HS1, Konica Minolta, Inc. Tokyo, Japan). An arthroscopic portal was created under ultrasound guidance, ensuring that the radial nerve running along the anterolateral aspect of the elbow joint was not damaged (Figures 2A, 2B). Similarly, another arthroscopic portal was created under ultrasound guidance on the anteromedial aspect of the elbow joint, ensuring that the median nerve and brachial artery were not damaged (Figure 2C). Although the joint cavity was narrow due to capsular thickening and shortening, an ultrasound image confirmed that the tip of the arthroscopy device was inserted between the bone and the joint capsule (Figure 2D).

**Figure 3:**
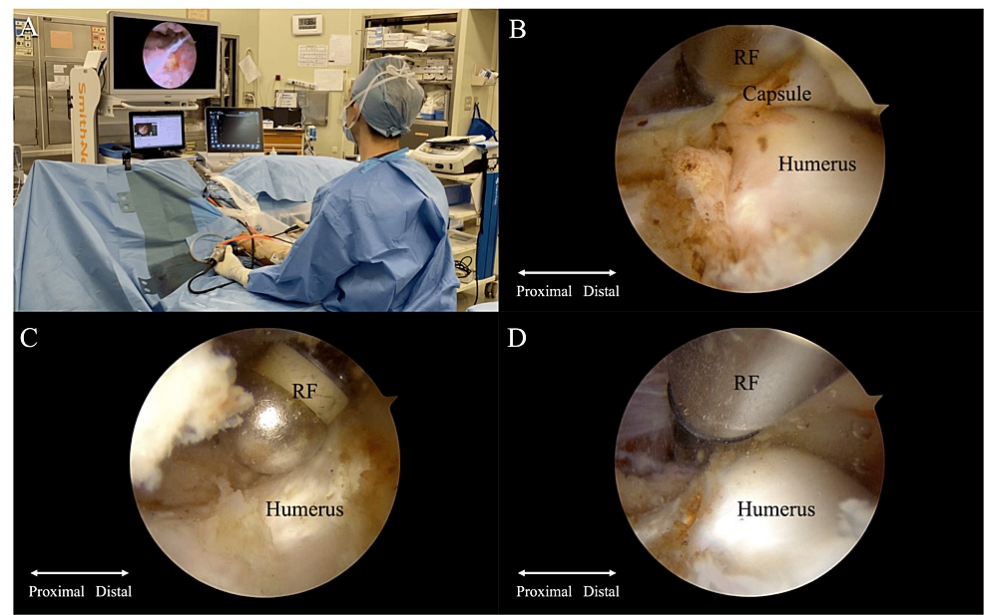
Arthroscopic capsule resection Elbow arthroscopy in the supine position (A). Under arthroscopic visualization, a radiofrequency device was used to ablate the joint capsule, and release it from the humerus (B-D). RF: radiofrequency device.

**Figure 4:**
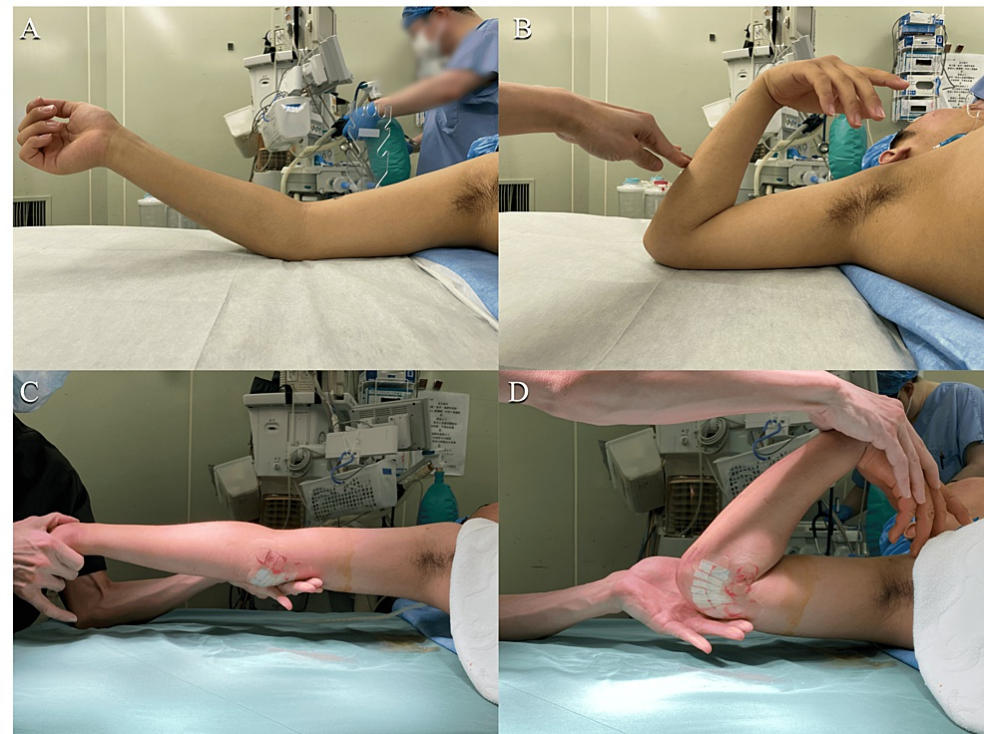
The elbow joint extension and flexion angle improved after the surgery (A, B) Pre operation. (C, D) Post operation.

Under arthroscopic visualization, a radiofrequency device was used to ablate the joint capsule and release it from the humerus (Figures 3A-3D). The elbow joint extension angle improved to 0 degrees, and the surgery was concluded (Figures 4A-4D).

There were no postoperative sensory or motor deficits suggestive of nerve damage. An arm sling was used to protect the affected area, and then the range of motion exercise was started the following day depending on the degree of pain. The patient did not complain of severe pain, and interventions like continuous nerve block were not required. A continuous passive motion was not used.

One month postoperatively, the range of motion in the elbow joint was largely preserved, with an extension of -8 degrees and a flexion of 135 degrees, and the patient returned to ice hockey. At seven months postoperatively, there was no deterioration in the range of motion, and the patient continues to compete.

## Discussion

The approach to elbow contractures from the anterior aspect of the elbow in the supine position is generally performed with direct visualization, but the problem is that it is highly invasive [7]. Therefore, arthroscopic surgery may be more useful to reduce invasiveness. However, the supine position is not commonly used for elbow arthroscopy [6,8].

In this case report, we describe a novel surgical technique for the management of elbow joint contracture, combining open and arthroscopic approaches in the supine position. The use of ultrasound-assisted arthroscopic surgery provided real-time visualization of internal tissues, including nerves and blood vessels [9,10]. This allowed for the safe creation of arthroscopic portals while minimizing the risk of damage to surrounding structures. Our technique demonstrates the feasibility of this approach, which eliminates the need for intraoperative position changes and big skin incisions.

## Conclusions

We introduced a novel surgical technique for elbow joint contracture that combines open and arthroscopic methods with ultrasound assistance in the supine position. By using ultrasound, we were able to safely create arthroscopic portals, making surgery possible without intraoperative position changes and big skin incisions. The patient progressed favorably without any complications. This surgical method is considered to be useful for elbow joint contracture.
